# Construction of a novel gene-based model for prognosis prediction of clear cell renal cell carcinoma

**DOI:** 10.1186/s12935-020-1113-6

**Published:** 2020-01-28

**Authors:** Zedan Zhang, Enyu Lin, Hongkai Zhuang, Lu Xie, Xiaoqiang Feng, Jiumin Liu, Yuming Yu

**Affiliations:** 1 Department of Urology, Guangdong Provincial People’s Hospital, Guangdong Academy of Medical Sciences, Guangzhou, China; 2 Shantou University Medical College, Shantou, China; 3 Department of Immunology, School of Basic Medical Science, Southern Medical University, Guangzhou, China

**Keywords:** Clear cell renal cell carcinoma (ccRCC), Differentially expressed genes (DEGs), Overall survival (OS), Risk score, Nomogram, TCGA, GEO

## Abstract

**Background:**

Clear cell renal cell carcinoma (ccRCC) comprises the majority of kidney cancer death worldwide, whose incidence and mortality are not promising. Identifying ideal biomarkers to construct a more accurate prognostic model than conventional clinical parameters is crucial.

**Methods:**

Raw count of RNA-sequencing data and clinicopathological data were acquired from The Cancer Genome Atlas (TCGA). Tumor samples were divided into two sets. Differentially expressed genes (DEGs) were screened in the whole set and prognosis-related genes were identified from the training set. Their common genes were used in LASSO and best subset regression which were performed to identify the best prognostic 5 genes. The gene-based risk score was developed based on the Cox coefficient of the individual gene. Time-dependent receiver operating characteristic (ROC) and Kaplan–Meier (KM) survival analysis were used to assess its prognostic power. GSE29609 dataset from GEO (Gene Expression Omnibus) database was used to validate the signature. Univariate and multivariate Cox regression were performed to screen independent prognostic parameters to construct a nomogram. The predictive power of the nomogram was revealed by time-dependent ROC curves and the calibration plot and verified in the validation set. Finally, Functional enrichment analysis of DEGs and 5 novel genes were performed to suggest the potential biological pathways.

**Results:**

PADI1, ATP6V0D2, DPP6, C9orf135 and PLG were screened to be significantly related to the prognosis of ccRCC patients. The risk score effectively stratified the patients into high-risk group with poor overall survival (OS) based on survival analysis. AJCC-stage, age, recurrence and risk score were regarded as independent prognostic parameters by Cox regression analysis and were used to construct a nomogram. Time-dependent ROC curves showed the nomogram performed best in 1-, 3- and 5-year survival predictions compared with AJCC-stage and risk score in validation sets. The calibration plot showed good agreement of the nomogram between predicted and observed outcomes. Functional enrichment analysis suggested several enriched biological pathways related to cancer.

**Conclusions:**

In our study, we constructed a gene-based model integrating clinical prognostic parameters to predict prognosis of ccRCC well, which might provide a reliable prognosis assessment tool for clinician and aid treatment decision-making in the clinic.

## Background

Renal cell carcinoma (RCC) ranks among the top ten cancer diagnoses worldwide, which account for 5% and 3% of all new cancer cases in males and females, respectively [1]. According to the latest data from the World Health Organization, there are more than 140,000 RCC–related deaths per year [2]. Among the RCC subtypes, clear-cell renal cell carcinoma (ccRCC) is the most common one and comprises the majority of kidney cancer deaths [3]. Therefore, identifying reliable prognostic tools for predicting the clinical outcomes and helping make decisions regarding observation, surgery, drug therapy and conservative options is obviously crucial for now.

Biomarkers used to predict overall survival (OS) can range from clinical parameters, endogenous substances and pathohistological characteristics of tumor to specific mutated gene. For example, the tumor node metastasis (TNM) classification system is most widely used to estimate prognosis and guide treatment in patients with cancer [4]. Besides, more and more single signature have been explored to predict the OS of ccRCC patients, such as CX3CR1 [5], miR-497 [6] and LncRNA CADM1-AS1 [7]. However, it is a challenge to predict survival of patients with ccRCC using single parameter by reason of the impact of wide variability of outcomes and genetic heterogeneity [8]. Thus, it is the best way to develop a comprehensive prognostic evaluation system including multiple biomarkers which can improve the predictive accuracy.

Nowadays, gene-based prognostic models containing other clinical parameters in predicting OS of cancer patients including ccRCC have been investigated numerously but they have not been widely accepted and exerted on the clinical practice [9–11]. Therefore, more novel prognosis-related genes could be uncovered by different bioinformatics analysis process and used to establish a more accurate prognostic models than conventional clinical parameters.

In this study, we constructed a model based on multiple prognostic-related genes and clinical parameters to predict OS of ccRCC patients. We screened the high-throughput sequence data from The Cancer Genome Atlas (TCGA) to explore differentially expressed genes and used the univariate Cox proportional hazards regression analysis, Least Absolute Shrinkage and Selection Operator method (LASSO) as well as best subset regression (BSR) to identify a five-gene group which got the lowest AIC value. The risk score was calculated through the multivariate cox coefficient multiplied by the expression of the gene. External validation was performed to verify the risk score model. Then the risk score and clinical parameters were combined together to construct a nomogram which was assessed by the calibration plot and time-dependent receiver operating characteristic curve (tROC) analysis. Furthermore, we did an internal validation to verify the model. Finally, functional enrichment analysis was performed to identify the potential biological pathways of the DEGs and five novel genes.

## Materials and methods

### Datasets sources and processing

Raw counts of RNA-sequencing data (level 3) and corresponding clinical information (Additional file 1: Table S1) from 533 KIRC and 78 paracancerous samples were obtained from The Cancer Genome Atlas (TCGA) dataset (https://portal.gdc.cancer.gov/) in April 2018, in which the method of acquisition and application complied with the guidelines and policies. Based on the requirement to the data integrality, patients that met the following criteria were excluded from subsequent analysis: (1) patients with survival time less than 30 days, (2) insufficient information of TNM, stage, grade, recurrence, age and gender. Finally, 504 tumor samples which were from different individuals and 71 paracancerous samples were selected from the dataset in this study. The patients (n = 504) were further randomly assigned to a training set and a testing set by a ratio of 7 to 3. Entrez IDs from gene expression data were converted to gene IDs by using a GTF file, which was downloaded from GENCODE (https://www.gencodegenes.org/). According to the selection criteria that gene was excluded if the sum of its expression level for each sample is less than 1, 19,651 protein-coding genes annotated by gene IDs above and were selected for further analysis.

Meanwhile, one microarray dataset GSE29609 which includes 39 KIRC patients with corresponding clinical information (Additional file 1: Table S1) was downloaded from GEO (http://www.ncbi.nlm.nih.gov/geo/) for external validation. It was performed on Agilent-012391 Whole Human Genome Oligo Microarray G4112A platform. The normalized expression matrix of microarray data could be directly download from the dataset. The probes were annotated by using the corresponding annotation files from the dataset as well. Then a principal component analysis (PCA) was used to detect whether the microarray dataset had the batch effect. The “sva” R package was used to eliminate the batch effect [12].

### Differential genes expression analysis of ccRCC

The raw count data of mRNA profile in ccRCC from TCGA dataset including tumor and paracancerous groups were normalized and quantile filtered by “voom” transformation and the differentially expressed genes (DEGs) were analyzed using the “limma” package of R software [13]. DEGs including significantly upregulated and downregulated genes were screened to subsequent analysis with an adjusted *p* value < 0.05 and absolute log2 fold change (FC) > 4.

### Selection and verification of prognosis-related genes

The raw counts of RNA-sequencing data were normalized with transcripts per million (TPM) method and using a log2-based transformation (log_2_TPM) for subsequent survival analysis.

Then this normalized expression data from the training set (n = 353) were used to build a panel of multi-gene signature to predict prognosis in ccRCC. Firstly, the expression data transformed by log_2_ TPM and the corresponding clinical information were used to screen out the prognosis-related genes using univariate Cox proportional hazards regression analysis (Hazard Ratio (HR) ≠ 1, p < 0.05). Then the prognosis-related genes (HR > 1, higher expression of genes indicate poor prognosis of patients) were intersected with the upregulated DEGs to obtain one set of candidate genes. The prognosis-related genes (HR < 1, lower expression of genes indicate poor prognosis of patients) were intersected with the downregulated DEGs to obtain another set of candidate genes. Finally, these two set of genes called overlapping candidate genes (OCGs) were used for subsequent analysis.

LASSO (Least Absolute Shrinkage and Selection Operator) regression was applied to construct a multi-gene signature with the OCGs for predicting prognosis in ccRCC using “glmnet” package of R software [14]. To improve the reliability and objectivity of analysis result, tenfold cross-validation was performed to identify the optimal lambda value that came from the minimum partial likelihood deviance.

Then the prognosis-related genes screened from LASSO algorithm with tenfold cross-validation was further analyzed in BSR, which is an exploratory model building regression analysis and can compare all possible created models based upon an identified set of genes. Supposed there were A prognosis-related genes (A = number) screened from LASSO algorithm. More detailed algorithm is summarized as follows:

1. k = 1, k = 2,…, k = A.
2. Chose k genes from A genes to construct models C (A, k), whose akaike information criterion (AIC) was calculated by means of “glmulti” package of R software [15].
3. According to the smallest AIC (sAIC) calculated above, C_sAIC_ (A, k) would be selected as the best optimal model consists of k genes.

However, taking into account of the feasibility of clinical work where the lesser number of the biomarkers in the model, the more advantage it gets in the clinic, the maximum value of k range was set to five [9, 16, 17]. Then patients from training set were divided into two groups according to the expression of every gene from C_sAIC_ (A, k) screened through BSR: high expression (log_2_TPM higher than the cutpoint, which determined by “survminer” package of R software [18]), and low expression (log_2_TPM lower than the cutpoint). Then KM curves as well as a log-rank test were implemented using R package “survival” [19] to show the relationship between expression of candidate genes and OS in ccRCC patients.

### Establishment and estimation of mulit-gene prognostic signature

The regression coefficients of 5 optimal prognostic genes were derived from the multivariate Cox proportional hazards regression model. Subsequently, a linear combination method was adopted to assemble expression level and coefficient of each gene to get a risk score formula, which is as follows:

$${\text{Risk score}} = \mathop \sum \limits_{i = 1}^{5} \beta_{i} *Exp_{i}$$

where Exp is the expression level of each prognostic gene, and β is the regression coefficient of it.

The patients in the training set were stratified into high-risk and low-risk groups based on the median risk score as the cutoff. The KM survival analysis with log-rank test were also used to compare the survival difference between above two groups. Univariate Cox proportional hazards regression analysis was performed to compare the prognostic power of the risk score and some clinical parameters including, T-stage, N-stage, M-stage, AJCC-stage, grade, gender, age, laterality and recurrence. Furthermore, we used multivariate Cox proportional hazards regression analysis to determine whether the risk score could be an independent prognostic factor in ccRCC patients based on risk levels. Other clinical parameters with statistically significant difference (p < 0.05) in univariate Cox proportional hazards regression were also incorporated in the analysis.

In order to explore the diagnostic capability of multi-gene prognostic signature in different levels of other clinical prognostic parameters, the KM curves were used to compare the difference of subgroups of AJCC-stage, grade, age, gender, laterality and recurrence, which were grouped by risk level for each sample in training set. Besides, tROC analysis was performed to compare the predictive accuracy of each gene and risk score.

### Validation of multi-gene prognostic signature

For internal and external validation, the testing set (n = 151), whole set (n = 504) and external validation set (n = 39) were used to validate the predictive capability and applicability of the multi-gene prognostic signature in ccRCC. In validation set, the risk score of each patient was calculated using the coefficients of 5 genes above. Then the patients were stratified into high-risk and low-risk groups by the median risk score from the training set. The KM survival analysis with log-rank test and tROC analysis were used to validate the multi-gene prognostic signature.

The image of immunohistochemistry (IHC) staining of the selected prognosis-related genes in normal tissue and ccRCC tissue were retrived from Human Protein Atlas online database (http://www.proteinatlas.org). Moreover, the mutation type of the finally selected prognosis-related genes was explored in cBioPortal (http://cbioportal.org).

### Construction and validation of gene prognostic nomogram

A composite nomogram was constructed based on all independent prognostic parameters screened by univariate and multivariate Cox proportional hazards regression analysis above to predict the probability of 1-year, 3-year and 5-year OS using “rms” package of R software [20].

The tROC curves were plotted to assess the predictive accuracy of independent prognostic parameters including AJCC-stage, risk level and gene prognostic nomogram using the R package “survivalROC” [21]. The area under the ROC curve (AUC) was calculated to make a comparison for discriminatory ability of above prognostic parameters. Then we used calibration curve to visualize the performance of the nomogram with the observed rates of training set at corresponding time points by a bootstrap method with 1000 resamples. The predicted and observed outcomes of the nomogram could be compared in the calibration curve while the 45° line represents the best prediction. The same methods were used in the testing set and the whole set to validate the results.

### Functional enrichment analysis of DEGs and prognosis-related genes

With the screened DEGs, gene ontology (GO) enrichment analysis and Kyoto Encyclopedia of Genes and Genomes (KEGG) pathways analysis were performed on the online tool-Metascape [22] (http://metascape.org/gp/index.html#/main/step1). A p-value of < 0.05 was considered as statistically significant.

As for the ultimate prognosis-related genes used for nomogram construction, Gene Set Enrichment Analysis (GSEA) was performed to identify the potential biological pathways. The whole set of 504 ccRCC samples were divided into two groups based on the median expression of each prognosis-related gene discussed above. Then GSEA software (v3.0, http://software.broadinstitute.org/gsea/) was conducted on JAVA 8.0 platform. The annotated gene set c2.cp.kegg.v6.2.symbols.gmt obtained from the Molecular Signatures Database (MSigDB) was chosen as the reference set to calculate Enrichment Score (ES) which estimated whether genes from prior defined gene set are enriched in high/low expression group of each prognosis-related gene or distributed randomly. The number of permutations was set to 1000. Gene size smaller than 15 or larger than 500 was excluded. A gene set was considered as a enriched group when the normalized p value < 0.05 and FDR score < 0.05 [23].

### Statistical analysis

The samples of tumor tissues were randomly divided into two groups using “sample” function of R software. Heatmap of DEGs was plotted using “pheatmap” R package [24] with zero-mean normalization. PCA was used to estimate batch effect and clustering result using “ggfortify” R package [25]. Two groups of boxplot were analyzed using Wilcoxon-test. For Kaplan–Meier curves, p-values and hazard ratio (HR) with 95% confidence interval (CI) were generated by log-rank tests and univariate Cox proportional hazards regression. All analytical methods above and R packages were performed using R software version 3.6.1 (The R Foundation for Statistical Computing, 2019). All statistical tests were two-sided. p < 0.05 was considered as statistically significant.

## Results

### Identification of DEGs

**Fig. 1:**
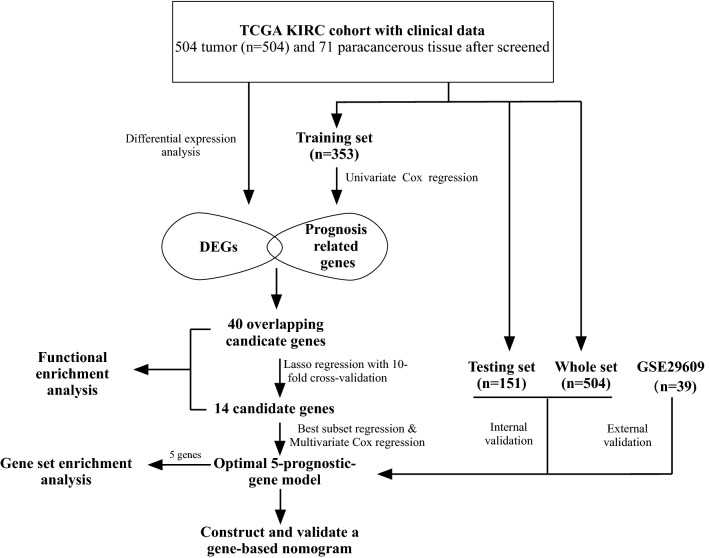
Flowchart of the whole study

**Fig. 2:**
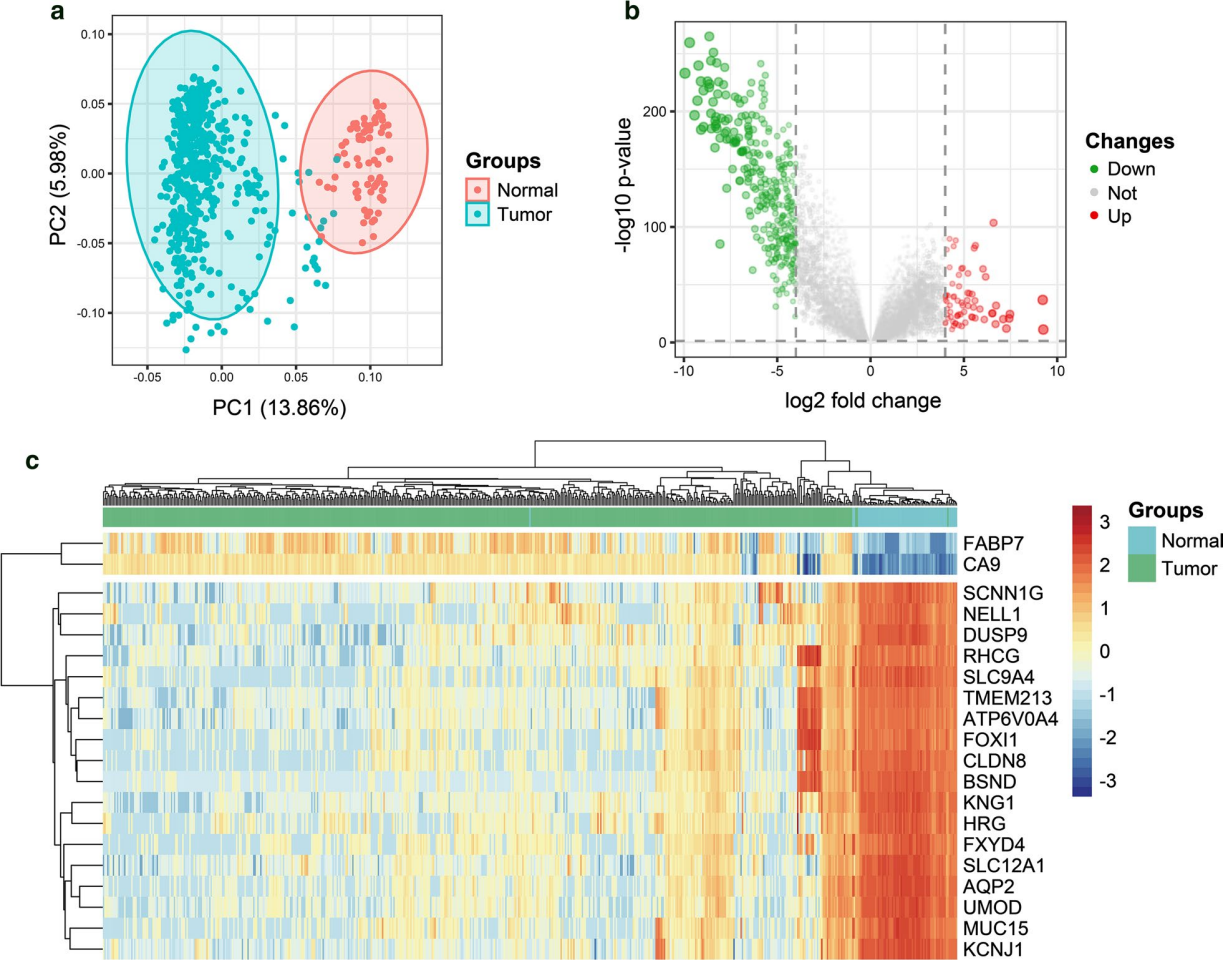
Data processing, screening of the DEGs. **a** A PCA plot of the data showing no batch effect in the TCGA KIRC dataset. Red nodes represent the normal cluster while blue nodes represent the tumor cluster. **b** Heatmap of top 20 DEGs in ccRCC. **c** Volcano plot of differentially expressed genes in ccRCC when compared with normal tissue. Red nodes represent the significantly up-regulated genes with logFC > 4 and p < 0.05. Green nodes represent the significantly down-regulated genes with logFC < -4 and p < 0.05. PCA, principal component analysis; TCGA, The Cancer Genome Atlas; DEGs, differentially expressed genes; ccRCC, clear cell renal cell carcinoma

The flowchart of our study is shown in Fig. 1. A total of 19,651 protein-coding genes were screened firstly from the raw counts of RNA-sequencing data from TCGA dataset. Then there was a significant difference in the level of transcript group between cancer and paracancerous tissue from PCA, whose figure showed that the clusters of tumor group were independent of normal group without obvious intersection (Fig. 2a). Subsequently, a total of 399 DEGs were identified, which included 71 upregulated and 328 downregulated genes (Fig. 2b, Additional file 2: Table S2). The heatmap of top 20 DEGs in ccRCC was shown as well (Fig. 2c).

### Screening and verification of prognosis-related DEGs

**Fig. 3:**
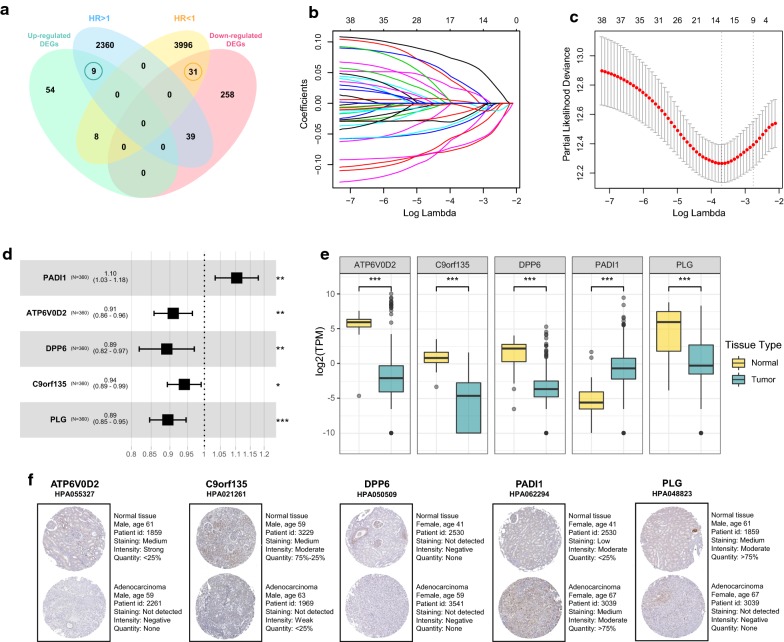
Identification of 5 significantly prognostic genes and their expression data in ccRCC. **a** Venn diagram of 40 OCGs. 9 upregulated genes with HR > 1. 31 downregulated genes with HR < 1. **b** LASSO coefficients profiles of 19651 protein-coding genes. **c** LASSO regression with tenfold cross-validation obtained 14 prognostic genes using minimum lambda value. **d** Multivariate Cox regression analysis of 5 prognostic genes from BSR. **e** Expression pattern of the five genes between tumor and normal kidney tissue. **f** IHC of the five genes in tumor and normal kidney tissue. *ccRCC* clear cell renal cell carcinoma, *OCG* overlapping candidate genes, *HR* hazard ratio, *LASSO* least absolute shrinkage and selection operator, *BSR* best subset regression, *IHC* immunohistochemistry. *p < 0.05, **p < 0.01, ***p < 0.001

According to the screening method and criteria discussed above, 2408 prognosis-related genes (HR > 1) and 4035 prognosis-related genes (HR < 1) were found totally in the training set (n = 353). Then the 2408 and 4035 prognosis-related genes were intersected with the 71 upregulated genes and 328 downregulated genes, respectively. Finally, 40 overlapping candidate genes (OCGs) were obtained, which included 9 DEGs with HR > 1 and 31 DEGs with HR < 1 (Fig. 3a, Additional file 3: Table S3).

To further identify the 40 OCGs that were significantly correlated with the prognosis of ccRCC patients, LASSO regression with tenfold cross-validation was performed to get the optimal lambda value that came from the minimum partial likelihood deviance (λ_min_ = 0.025), which was related with 14 genes in DEGs that significantly associated with OS (Fig. 3b, c). Then BSR analysis directly identified the optimal 5-prognostic-gene model which was selected as with the lowest AIC value, namely PADI1 (Peptidyl Arginine Deiminase 1), ATP6V0D2 (ATPase H + Transporting V0 Subunit D2), DPP6 (Dipeptidyl Peptidase Like 6), C9orf135 (Chromosome 9 Open Reading Frame 135), PLG (Plasminogen).

**Fig. 4:**
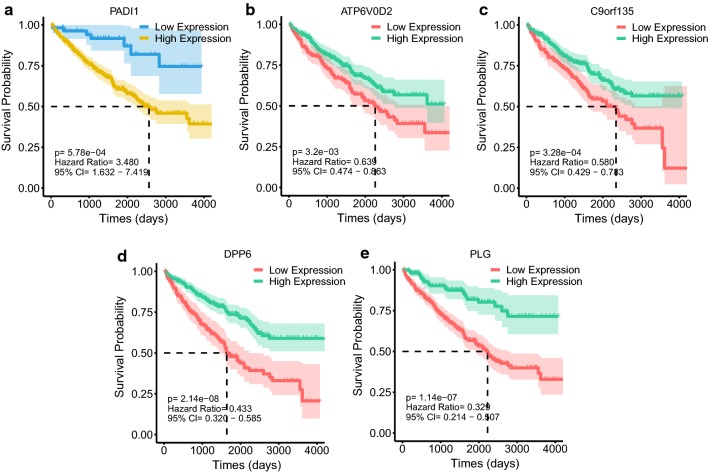
Kaplan–Meier survival analysis of PADI1, ATP6V0D2, C9orf135, DPP6, and PLG

The median of 5-gene expression quantity was regarded as a cutoff to partition the training set samples into high expression and low expression group respectively, which were used to perform survival analysis. Overexpression of PADI1 and low expression of ATP6V0D2, DPP6, C9orf135 and PLG were associated with the poor prognosis of ccRCC patients (Fig. 4) (p < 0.05). The KM survival curves of other 9 genes including AHNAK2, CXCL13, HSF4, PPDPFL, TMEM45B, SVOPL, SLC34A1, PIGR and CPNE6 used in BSR analysis were shown in Additional file 4: Figure S1.

### Expression profiles, IHC and genetic alteration of prognosis-related genes

Then the expression profiles of the five genes between tumor and normal tissue shown in Fig. 3E which indicated that PADI1 was significantly upregulated in ccRCC while ATP6V0D2, DPP6, C9orf135 and PLG were significantly downregulated when compared with normal tissue (p < 0.001). Moreover, Human Protein Atlas database was used to validate the protein expression of the five genes. However, no difference was found for DPP6 protein expression (Fig. 3f). Furthermore, the association between the expression levels of the five genes and histopathological information including AJCC-stage (Additional file 5: Figure S2A), nodal metastasis (Additional file 5: Figure S2B) and tumor grade (Additional file 5: Figure S2C) was explored on UALCAN [26] (http://ualcan.path.uab.edu/analysis.html) in TCGA samples. Among the five genes in ccRCC, ATP6V0D2, DPP6, PADI1 and PLG were significantly associated with AJCC-stage (p < 0.05); ATP6V0D2, C9orf135, DPP6 and PLG were significantly associated with nodal metastasis (p < 0.05); ATP6V0D2, DPP6 and PLG were significantly associated with tumor grade (p < 0.05). Finally, the type of genetic alteration type of five genes was searched in cBioPortal database including not only TCGA but also other four databases shown in Additional file 6: Figure S3. Amplification was common in ATP6V0D2, DPP6 and C9orf135, while deep deletion was common in PADI1, C9orf135 and PLG in ccRCC patients.

### Establishment and estimation of the five-gene prognostic signature

**Fig. 5:**
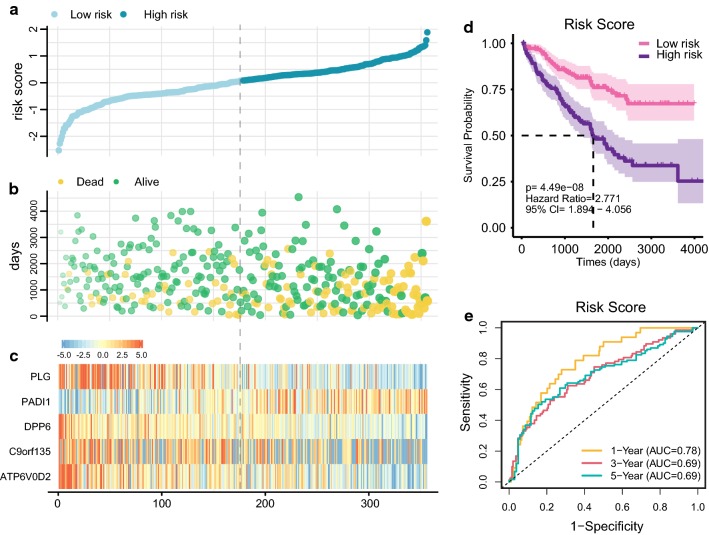
Prognostic analysis of five-gene signature in the training set. The dotted line represented the median risk score and divided the patients into low- and high-risk group. **a** The curve of risk score. **b** Survival status of the patients. More dead patients corresponding to the higher risk score. **c** Heatmap of the expression profiles of the five prognostic genes in low- and high-risk group. **d** Kaplan–Meier survival analysis of the five-gene signature. **e** Time-dependent ROC analysis the of the five-gene signature. *ROC* receiver operating characteristic

**Fig. 6:**
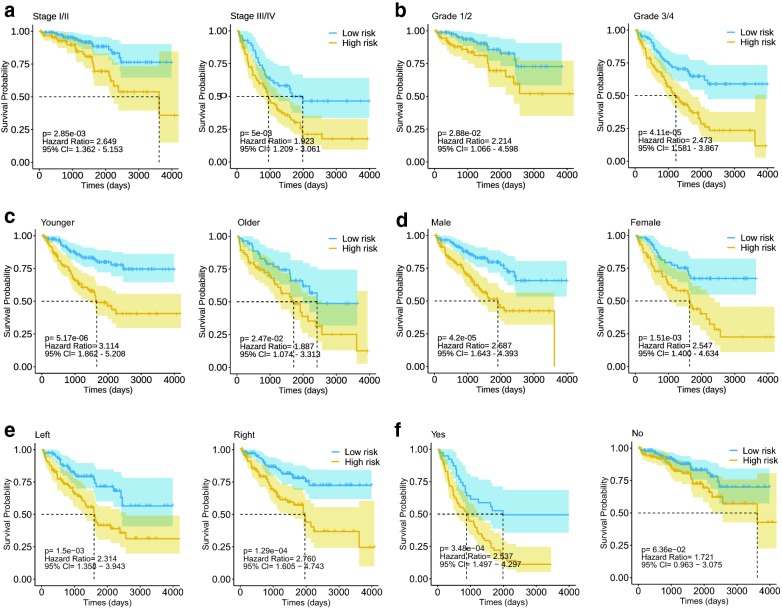
Kaplan–Meier survival analysis of the five-gene risk score level in different subgroups including stage I/II, stage III/IV (**a**), grade 1/2, grade 3/4 (**b**), younger than 65 years old, older than 65 years old (**c**), male, female (**d**), left tumor, right tumor (**e**) and with or without recurrence (**f**)

Multivariate Cox proportional hazards regression analysis was performed on 5 prognostic genes to determine whether each gene could exhibit a significant prognostic value for ccRCC patients (Fig. 3d). Therefore, the five gene-based risk score was constructed based on their Cox coefficients: risk score = 0.09862331*Exp_(PADI1)_ − 0.09526638*Exp_(ATP6V0D2)_ − 0.11493839*Exp_(DPP6)_ − 0.06144184*Exp_(C9orf135)_ − 0.11164739*Exp_(PLG)._ Then the risk score of every patient was calculated, among which we used “survminer” R package to obtain the median cut-off point and divided the patients into the high-risk group (n = 176) and low-risk group (n = 177) (Fig. 5a). Figure 5b shows the survival status of all patients in the training group and Fig. 5c presents the heatmap of 5 prognostic genes. The KM survival curves showed that the high-risk group had worse OS compared with the low-risk group (Fig. 5d). Besides, we performed risk stratification in patients with AJCC-stage, grade, gender, age, laterality and recurrence, and did the KM survival analysis (Fig. 6). The patients with high-risk scores had worse OS than the patients with low-risk scores in stage I/II (p < 0.01), stage III/IV (p < 0.01), grade 1/2 (p = 0.0288), grade 3/4 (p < 0.01), younger (p < 0.01), older (p = 0.0247), male (p < 0.01), female (p < 0.01), left side of tumor (p < 0.01), right side of tumor (p < 0.01) and recurrence (p < 0.01). Moreover, the five-gene prognostic signature showed larger AUC values in a time-dependent ROC analysis (Fig. 5e) compared with each gene above (Additional file 7: Figure S4), which meant that multi-gene model had better prediction ability in 1-year, 3-year and 5-year OS.

### Internal and external validation of five-gene prognostic signature

**Fig. 7:**
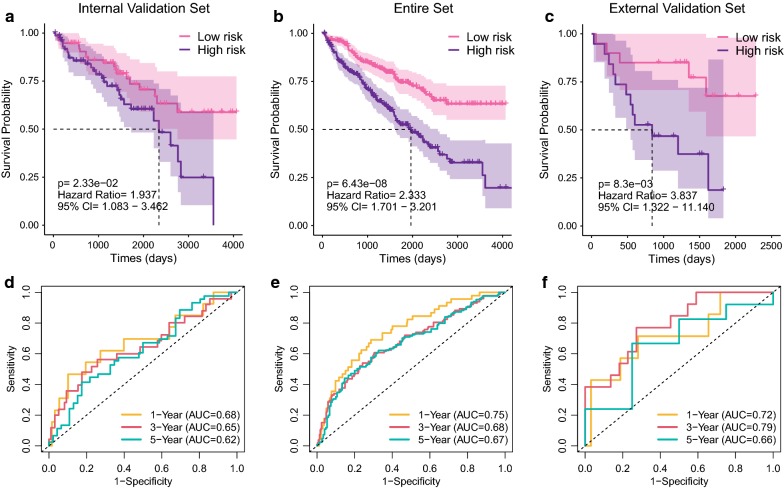
Validation of the five-gene signature. GSE29609 was regarded as the external validation set. Kaplan–Meier survival analysis of the five-gene signature in internal validation set (**a**), the whole set (**b**) and external validation set (**c**). Time-dependent ROC analysis of the five-gene signature in internal validation set (**d**), the whole set **(e**) and external validation set (**f**). ROC, receiver operating characteristic

To verify the predictive value of the five-gene prognostic signature, we used the internal validation set (n = 151), the whole set (n = 504) and GSE29609 as the external validation set (n = 39) to assess the findings from the training set. PCA of GSE29609 showed that it had significant batch effect (Additional file 8: Figure S5A). After using the “sva” R package to, the batch effect was eliminated and the external dataset could be used more accurately for subsequent analysis (Additional file 8: Figure S5b, c). Consistent of the results in the training set, the KM curves of the three testing sets showed that the high-risk groups had worse prognosis than the low-risk groups (Fig. 7a–c). Time-dependent ROC analysis showed that AUC for 1-year, 3-year and 5-year OS of the internal validation set, the whole set and the external validation set were 0.68, 0.65, 0.62, 0.75, 0.68, 0.67, 0.72, 0.79, 0.66, respectively (Fig. 7d–f). To sum up, the five-gene prognostic signature performed well in prediction of OS of ccRCC patients.

### Construction and validation of the gene-based nomogram

**Fig. 8:**
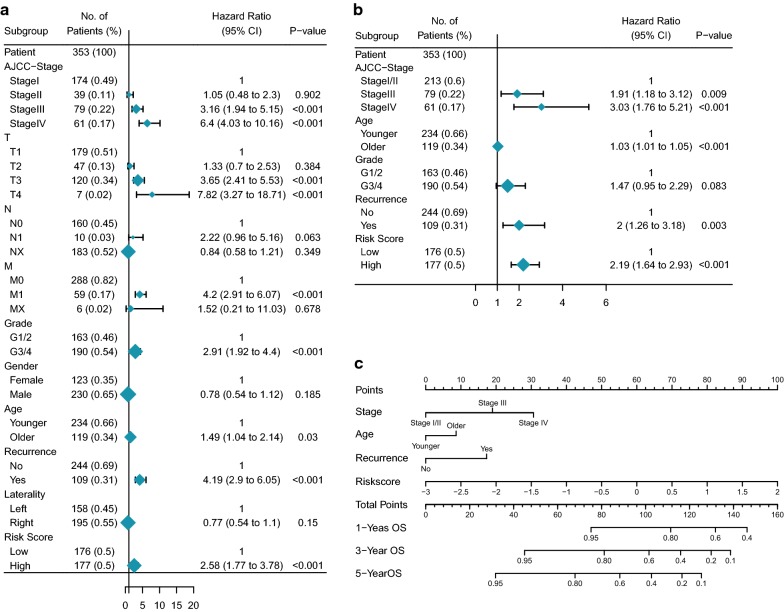
Identifying the independent prognostic parameters and construction of gene-based prognostic model. **a** Forrest plot of univariate Cox regression analysis in ccRCC. **b** Forrest plot of multivariate Cox regression analysis in ccRCC. **c** Nomogram integrated five gene-based risk score, AJCC-stage, grade and age

**Fig. 9:**
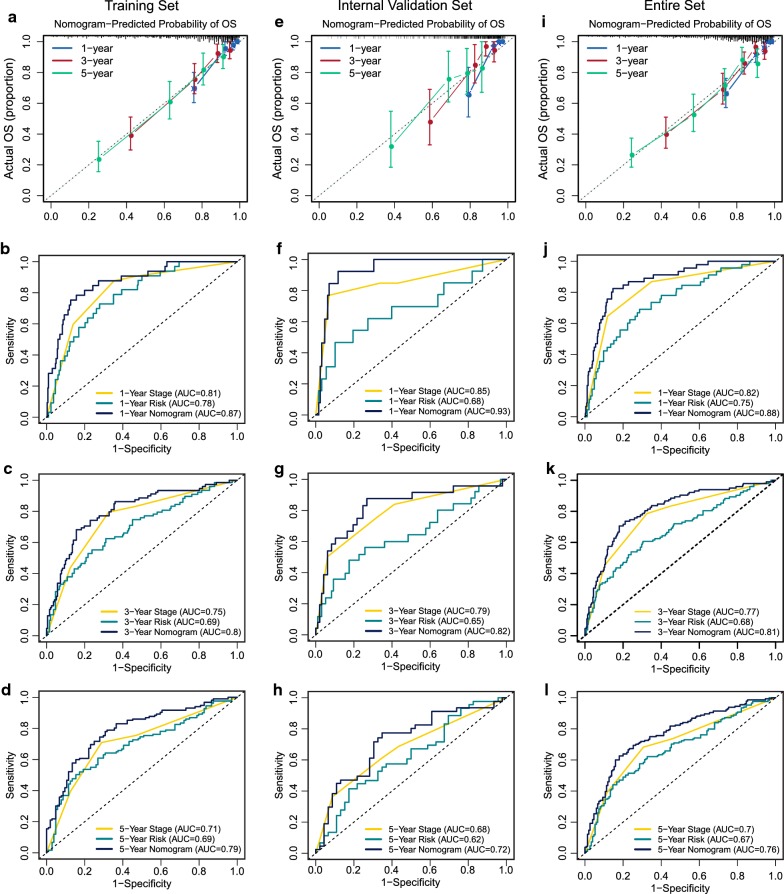
Performance of gene-based nomogram in predicting survival probability and comparison of the predictive power among gene-based nomogram, risk score and AJCC-stage. The calibration plot of the nomogram for agreement test between 1-, 3- and 5-year OS prediction and actual outcome in the training set (**a**), the internal validation set (**e**) and the entire set (**i**). The time-dependent ROC curves of the nomogram, risk score and AJCC-stage in 1-, 3- and 5-year OS prediction in the training set (**b**–**d**), the internal validation set (**f**–**h**) and the entire set (**j**–**l**). *OS* overall survival, *ROC* receiver operating characteristic

After analyzed by the univariate and multivariate Cox proportional hazards regression methods, the five-gene prognostic signature with other clinical parameters, such as AJCC-stage, age and recurrence, could be independent prognostic variables of the OS in the training set (Fig. 8a, b). In order to establish a more reliable predictive method for clinical practice, we constructed a compound nomogram integrating the risk score, AJCC-stage, age and recurrence to predict 1-year, 3-year and 5-year OS of ccRCC patients (Fig. 8c). The presentation of calibration plot for patient survival prediction demonstrated that the predicted outcome of 5-gene prognostic nomogram showed good agreement to the actual outcome (Fig. 9a). The AUC value of 1-year, 3-year and 5-year OS of nomogram was larger than that of risk score and AJCC-stage, which suggested that the 5-gene prognostic nomogram may be the best performance in predicting OS (Fig. 9b–d).

To confirm the predictive value of the 5-gene prognostic nomogram, we used an internal validation set (151) and the whole set (n = 504) to test the findings above. The calibration plot showed good agreement between the predicted and actual outcome of 1-year, 3-year and 5-year OS of the nomogram in the internal validation set and the whole set as the same as that in the training set (Fig. 9e, i). Time-dependent ROC curves of the risk score, AJCC-stage and 5-gene based nomogram were compared with each other (Fig. 9f–h, j–l). They showed that the nomogram no matter in which set had better prediction than AJCC-stage and risk score in 1-year, 3-year and 5-year OS.

### Identification of biological pathways of DEGs and five prognostic genes

**Fig. 10:**
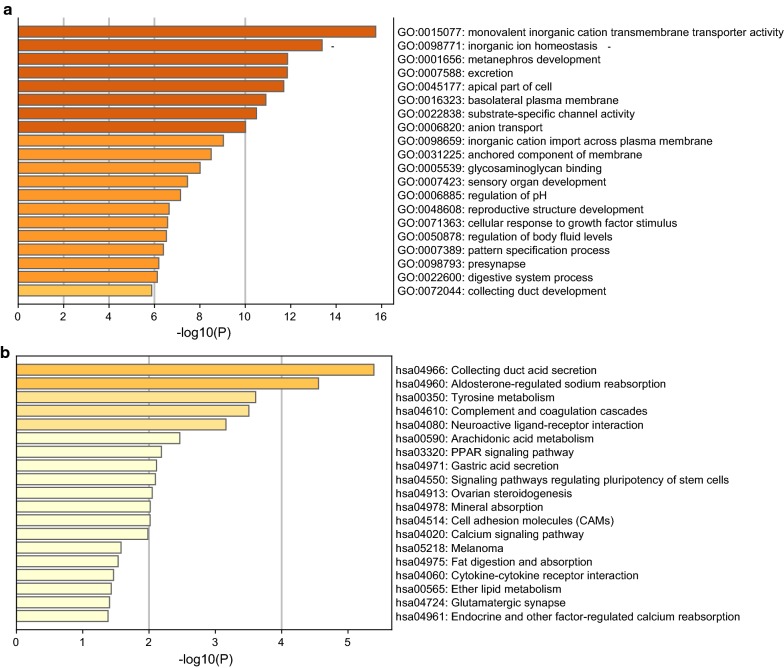
Functional enrichment analysis of 399 DEGs. The more genes enriched in the terms, the darker the color. **a** Top 20 of GO enrichment analysis of the DEGs. **b** Top 20 of KEGG enrichment analysis of the DEGs. *DEGs* differentially expressed genes, *GO* gene ontology, *KEGG* Kyoto Encyclopedia of Genes and Genomes

GO and KEGG enrichment analysis were used to identify the biological function of 399 DEGs. In GO biological analysis, the DEGs were enriched in monovalent inorganic cation transmembrane transporter activity, basolateral plasma membrane, anchored component of membrane and cellular response to growth factor stimulus, etc. (Fig. 10a, Additional file 9: Table S4). The network of GO biological process was also shown. In the diagram, different color represents the different biological process (Additional file 10: Figure S6A) and the degree of color means the counts of enriched genes, in which the darker the color, the more genes were enriched in corresponding process (Additional file 10: Figure S6B). In KEGG pathway analysis, PPAR signaling pathway, melanoma, cell adhesion molecules (CAMs) and other biological pathways were identified for DEGs (Fig. 10b, Additional file 11: Table S5).

**Fig. 11:**
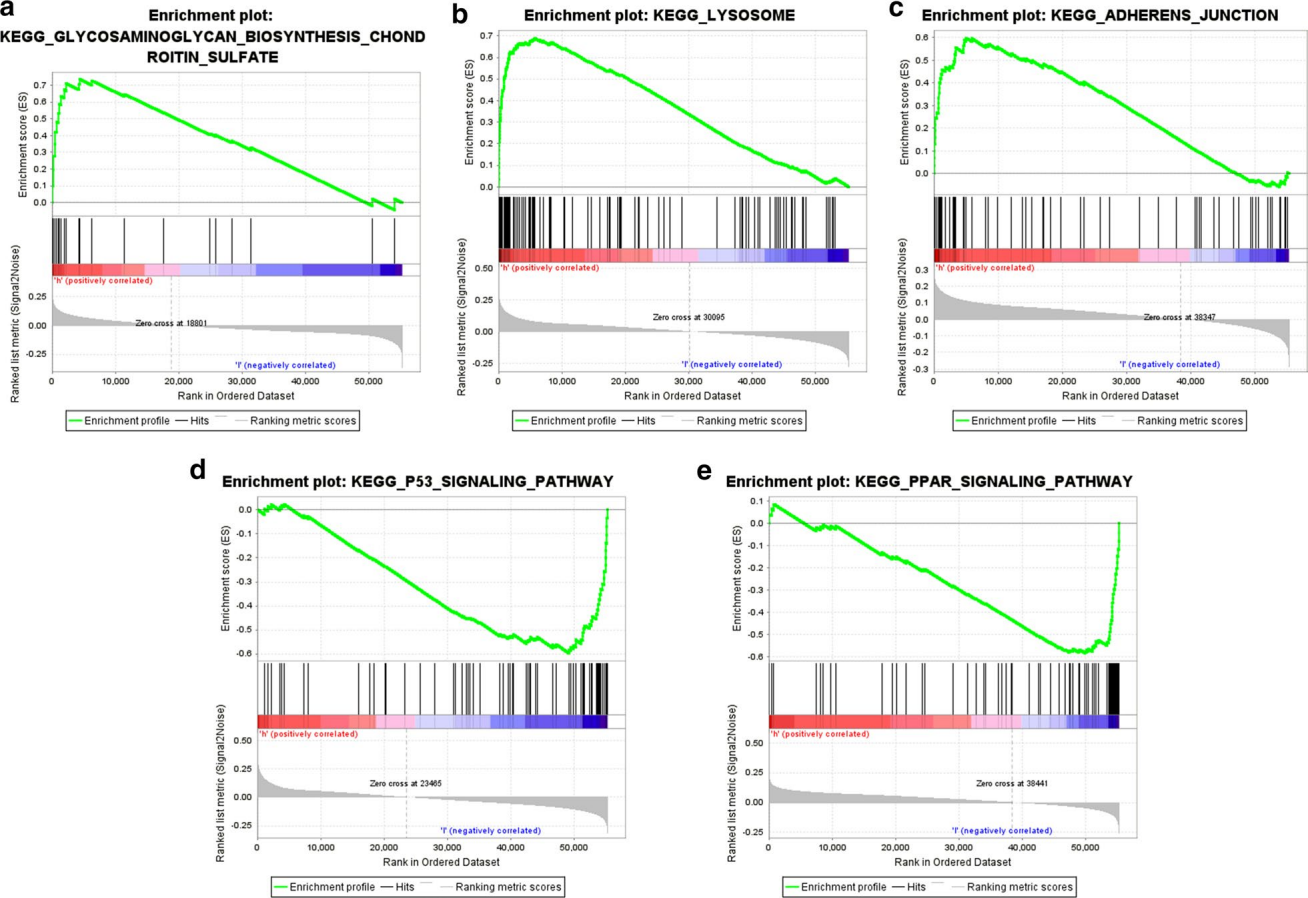
GSEA associated with the five genes expression. The gene set “GLYCOSAMINOGLYCAN_BIOSYNTHESIS_CHONDROTIN_SULFATE” (**a**), “LYSOSOME” (**b**), “ADHESION_JUNCTION” (**b**), and “GLYCOSAMINOGLYCAN_BIOSYNTHESIS_CHONDROTIN_SULFATE” (**c**) were enriched in ccRCC samples with highly expressed PADI1, DPP6 and ATP6V0D2, respectively. The gene set “P53_SIGNALING_PATHWAY” (**d**) “PPAR_SIGNALING_PATHWAY” (**e**) and were enriched in ccRCC samples with lowly expressed PLG and C9orf135, respectively

GSEA was performed to identify the potential biological processes of the five prognostic genes. Results revealed that the samples with the overexpression of ATP6V0D2, DPP6 and PADI1were enriched in lysosome, adhesion junction and glycosaminoglycan biosynthesis-chondroitin sulfate aspects respectively. The samples with the low expression of C9orf135 and PLG were enriched in the PPAR signaling pathway and p53 signaling pathway respectively (Fig. 11).

## Discussion

During the last two decades, the incidence of renal cell carcinoma significantly increased and the mortality was not promising [2, 27]. Identifying effective prognostic biomarkers to construct good prognostic tools to predict the survival of ccRCC patients is the advisable choice applied in the clinical practice. At present, the TMN staging system is commonly used to predict the prognosis of ccRCC patients [28]. But as discussion above, single clinical parameter has poor power of prognosis prediction. Thus, combining other prognostic parameters would be the best way to improve the accuracy of prediction.

In our current study, the DEGs were identified firstly from ccRCC and normal tissue and were found to be principally enriched in basolateral plasma membrane, anchored component of membrane, PPAR signaling pathway and cell adhesion molecules (CAMs). Then the intersected genes between DEGs and prognosis-related genes sifted out from univariate Cox regression methods in the training set were used in LASSO regression with tenfold cross-validation and BSR to screened out five novel DEGs (PADI1, ATP6V0D2, DPP6, C9orf135, PLG), where the order as well as the content of the screening methods were not all the same as the most research.

To the best of our knowledge, there has not been any study using the screening methods like ours to identify the upregulated DEGs with HR > 1 and downregulated DEGs with HR < 1. The method can exclude some situations such as upregulated DEGs with HR < 1 and downregulated DEGs with HR > 1, which are not practical in clinical practice. The five novel genes are significantly related to the OS of ccRCC patients. While PLG, DPP6, ATP6V0D2, C9orf135 are negative prognostic genes, PADI1 is a positive prognostic gene. PLG plays an important role in tissue remodeling during development, physical injury, inflammation and carcinogenesis. It can help degrade the extracellular matrix with other matrix metalloproteases, such as collagenases, gelatinases and stromelysins, which all serve a vital character in cancer invasion, especially in lung and breast cancer [29, 30]. However, PLG is not only a pro-tumorigenic factor but also an anti-tumorigenic factor due to the fact that proteolysis of PLG can release angiotensin, which will function against cancer progression [31]. This may explain the result that the expression level of PLG in ccRCC samples was lower than that in adjacent normal tissue in our study, which meant the low expression of PLG was important for ccRCC progression. In addition to our results, downregulation of PLG in ccRCC was confirmed by Schrödter et al. who screened the DEGs using a microarray and qPCR [32]. PLG was also screened as a hub gene in some research, which suggested it might play a major role in ccRCC [33, 34]. Worse OS of ccRCC patients associated with low expression of PLG was verified by Wang et al. using UALCAN [34]. Our GSEA analysis showed that low expression of PLG also probably negatively mediates p53 signaling pathway to promote ccRCC progression. DPP6 is known as a protein participating in modulating A-type potassium channels in somatodendritic compartments of neurons, which plays a role in synaptic plasticity [35]. Nevertheless, recent research has found that DPP6 could regulate various biological functions, maintain cell-specific phenotype and dysregulated expression of DPP6 would result in carcinogenesis [36, 37]. It was reported that DPP6 was down-regulated in acute myeloid leukemia and melanoma but up-regulated in colon cancer, which was probably caused by hyper- and hypomethylation, respectively [38–40]. In ccRCC, Song et al. also figured out that DPP6 was a downregulated gene in ccRCC samples compared with normal tissue by analyzing GEO and TCGA databases [41]. However, there are few studies regarding the role of DPP6 in ccRCC at present. PADI1 belongs to the peptidyl arginine deiminases family consisting of five family members (PADI1-4 and PADI6) in human. They catalyze the process of citrullination modification of proteins [42]. When the process is upregulated, it would disturb the stability of proteins and caused DNA damages, which is associated with carcinogenesis involved in the stomach, the liver, the large intestine, oral squamous cell carcinoma and so on [42–44]. Interestingly, overexpression of PADI driven by MZF1 and Sp1/Sp3 binding to the promoter region can citrullinate PKM2 and stimulate glycolysis in cancer cells [45, 46]. However, to the best of our knowledge, the specific correlation between PADI1 and ccRCC remains ill-defined. ATP6V0D2 is a gene encoding H^+^ transporting protein in the plasm membrane of cells, especially osteoclasts [47]. When ATP6V0D2 is downregulated, it will dysregulate the intracellular and extracellular acidic environment. Some research suggests that a high intracellular pH and a low extracellular pH will give cancer cells a competitive advantage over normal cells for growth [48]. But the specific correlation of ATP6V0D2 dysregulation and tumor acidity remains uncertain. Downregulated ATP6V0D2 probably functions through increasing HIF-2α expression produced by macrophage to enhance tumor vascularization and growth [49]. Previous studies showed that an elevated expression of ATP6V0D2 was found in stomach cancer specimens, whereas the expression was reduced in the colorectal and renal cancer specimens, which confirmed our findings [50, 51]. But so far, as for the specific mechanism between ATP6V0D2 and ccRCC, there has been no research reported yet. C9orf135, chromosome 9 open reading frame 135, encodes a membrane-associated protein whose expression is related to pluripotency in human embryonic stem cells (hESC). The expression of C9orf135 is regulated by OCT4 and SOX2 and decreases during hESC differentiation [52]. However, the role of C9orf135 has not been widely characterized in cancer. Ye et al. reported that its expression was downregulated in nasopharyngeal carcinoma [53]. Our GSEA suggests that low expression of C9orf135 probably promote ccRCC formation through affecting PPAR signaling pathway. Taken together, we revealed that the correlation between the expression level of the novel five genes and the OS of ccRCC patients; meanwhile, GSEA was also performed to identify the potential biological pathways of the novel five genes in ccRCC formation and progression. Due to the activity of five genes on carcinogenesis and the significant relevance to the prognosis of ccRCC patients, probably they can function as novel cancer biomarkers if the more details of their specific roles playing in ccRCC are explored widely and deeply.

After identifying the five prognostic genes, five-gene prognostic signature was developed and investigated for its prognostic value in ccRCC patients. The patients in high-risk groups showed significantly poorer prognosis than the patients in low-risk group. Moreover, the prediction of 5-gene prognostic signature could be used in different subgroups such as stage I/II, stage III/IV, grade 1/2, grade 3/4, male, female, younger (≤ 65 years old), older (≥ 65 years old), left and right site and recurrence group. There was significantly different prognosis between high-risk and low-risk level in these subgroups and all high-risk groups had worse OS than that of low-risk groups, which meant that the novel gene model could be used to stratify ccRCC patients into high-risk and low-risk group in these subgroups and help clinician choose wiser clinical decisions.

Then the univariate and multivariate Cox regression analysis showed that the five-gene prognosis signature could be an independent factor to evaluate the prognosis. Internal and external validation were also conducted to confirm its predictive value. Further, the time-dependent ROC analysis of each gene was performed and the results showed that the sensitivity and specificity of single parameter was poorer than that of five-gene prognostic signature, which suggested that the predictive power of multi variables would perform much better. However, the AUC of five-gene prognostic signature for 1-year, 3-year and 5-year OS showed a little bit smaller than that of AJCC-stage in three set (Fig. 9). In order to improve the ability to prognosis prediction of five-gene prognostic signature, a highly accurate predictive nomogram was constructed integrating the risk score and conventional clinical prognostic parameters including AJCC-stage, age and recurrence, all of which were verified as an independent prognostic factor using univariate and multivariate Cox proportional hazards regression analysis for the OS of ccRCC patients. It could be used to predict the individual 1-, 3- and 5-year OS probability specifically according to the risk score and other conventional clinical prognostic parameters. Then its time-dependent ROC survival analysis in the three sets revealed that it presented the best power of 1-, 3- and 5-year OS prediction compared with that of risk score system and AJCC-stage (Fig. 9). Very perfect agreement was observed in the calibration plot of our nomogram in the training set between the predicted and observed outcomes. Satisfied agreement was also seen in the internal validation and the whole set. Therefore, our five-gene based prognostic nomogram may aid clinician in predicting the survival outcome of ccRCC patients and provide the reference for therapy guidance than single conventional clinical parameter. Besides, to some extent, based on the hints about the drastically clinical significance of these five prognostic genes from our study, we think we provide the necessity of following functional experiment exploration.

However, several limitations in our study should be acknowledged. Firstly, our study only focused on the large-scale mRNA sequencing data from TCGA platform. Other types of data like single nucleotide polymorphisms (SNP), copy number variation (CNV) and DNA methylation are provided by the public dataset. If possible, five novel biomarkers could be analyzed further to see whether their expression level is related to mutation types above. Secondly, the significantly difference of protein expression level of the five genes between tumor and normal tissues could be detected in TCGA database, where patients are mainly Asian and White. More public database or experiment needs to be explored whether their expression level is geographically different. Thirdly, our study provides the evidence that five novel genes are significantly related to the survival of ccRCC patients and possibly become therapeutic targets for precision medicine in the future, which was analyzed through data mining merely. Functional experiment for revealing their roles in cancers is valuable and crucial.

## Conclusion

In our current study, we identified five novel prognostic DEGs from publicly available data and constructed a five-gene based prognostic nomogram which contained other clinical prognostic parameters using methodologically reasonable bioinformatics analysis to predict 1-year, 3-year and 5-year OS of ccRCC patients, whose power of prediction is better than that of conventional AJCC-stage. In other words, the five genes could be potential biomarkers in ccRCC and relevant gene-based nomogram could potentially be used in clinical practice for predicting the individual survival rate and promoting the selection of individual treatment options of ccRCC patients.

## Supplementary information

**Additional file 1: Table S1.** Clinical information of TCGA KIRC cohort and GSE39609 cohort.

**Additional file 2: Table S2.** Differentially expressed genes (DEGs) with an adjusted p-value < 0.05 and absolute log_2_ fold change (FC) > 4.

**Additional file 3: Table S3.** 40 overlapping candidate genes.

**Additional file 4: Figure S1.** Kaplan–Meier survival analysis of nine prognosis-related genes used in BSR. BSR, best subset regression.

**Additional file 5: Figure S2.** The expression pattern of the five prognosis-related genes in different AJCC-stage (A), node metastasis state (B) and grade (C) from UALCAN TCGA ccRCC samples. TCGA, The Cancer Genome Atlas.

**Additional file 6: Figure S3.** The type of gene alteration among 628 patietns/696 samples in 3 publicly datasets including TCGA from cBioportal. 23 (3%) patients have gene alternation.

**Additional file 7: Figure S4.** Time-dependent ROC analysis of the five prognosis-related genes in 1-, 3- and 5-year OS prediction. A C9orf135. B DPP6. C PLG. D ATP6V0D2. E PADI1. ROC, receiver operating characteristic.

**Additional file 8: Figure S5.** PCA plot of GSE29609 dataset from GEO. A PCA plot shows that there are two clusters in the dataset, which means the dataset has batch effect. B Colorized the two clusters. C PCA plot of the data after normalization. GEO, Gene Expression Omnibus; PCA, principal component analysis.

**Additional file 9: Table S4.** Gene ontology (GO) enrichment analysis of 399 DEGs.

**Additional file 10: Figure S6.** Network of GO enriched terms. (A) The color represents the GO terms. (B) The color represents the p-value. The more genes enriched in the terms, the darker the color. Go, Gene ontology.

**Additional file 11: Table S5.** Kyoto Encyclopedia of Genes and Genomes (KEGG) pathways analysis of 399 DEGs.

## Data Availability

The datasets generated and analyzed during the current study are available in the The Cancer Genome Atlas (TCGA), https://portal.gdc.cancer.gov/, and Gene Expression Omnibus, http://www.ncbi.nlm.nih.gov/geo/.

## Abbreviations

ccRCC: clear cell renal cell carcinoma
TCGA: The Cancer Genome Atlas
GEO: Gene Expression Omnibus
DEGs: differentially expressed genes
OCGs: overlapping candidate genes
KM: Kaplan–Meier
VST: variance stabilizing transformation
BSR: best subset regression
LASSO: Least Absolute Shrinkage and Selection Operator
OS: overall survival
FDR: false discovery rate
FC: fold change
HR: hazard ratio
CI: confidence interval
AJCC: the American Joint Committee on Cancer
ROC: receiver operating characteristic
GSEA: gene set enrichment analysis
GO: gene ontology
KEGG: Kyoto Encyclopedia of Genes and Genomes
