# Supplementary figures and images for: Construction of a novel gene-based model for prognosis prediction of clear cell renal cell carcinoma
Source: Cancer Cell Int. 2020 Jan 28;20:27. doi: 10.1186/s12935-020-1113-6 (PMC6986036; doi:10.1186/s12935-020-1113-6)

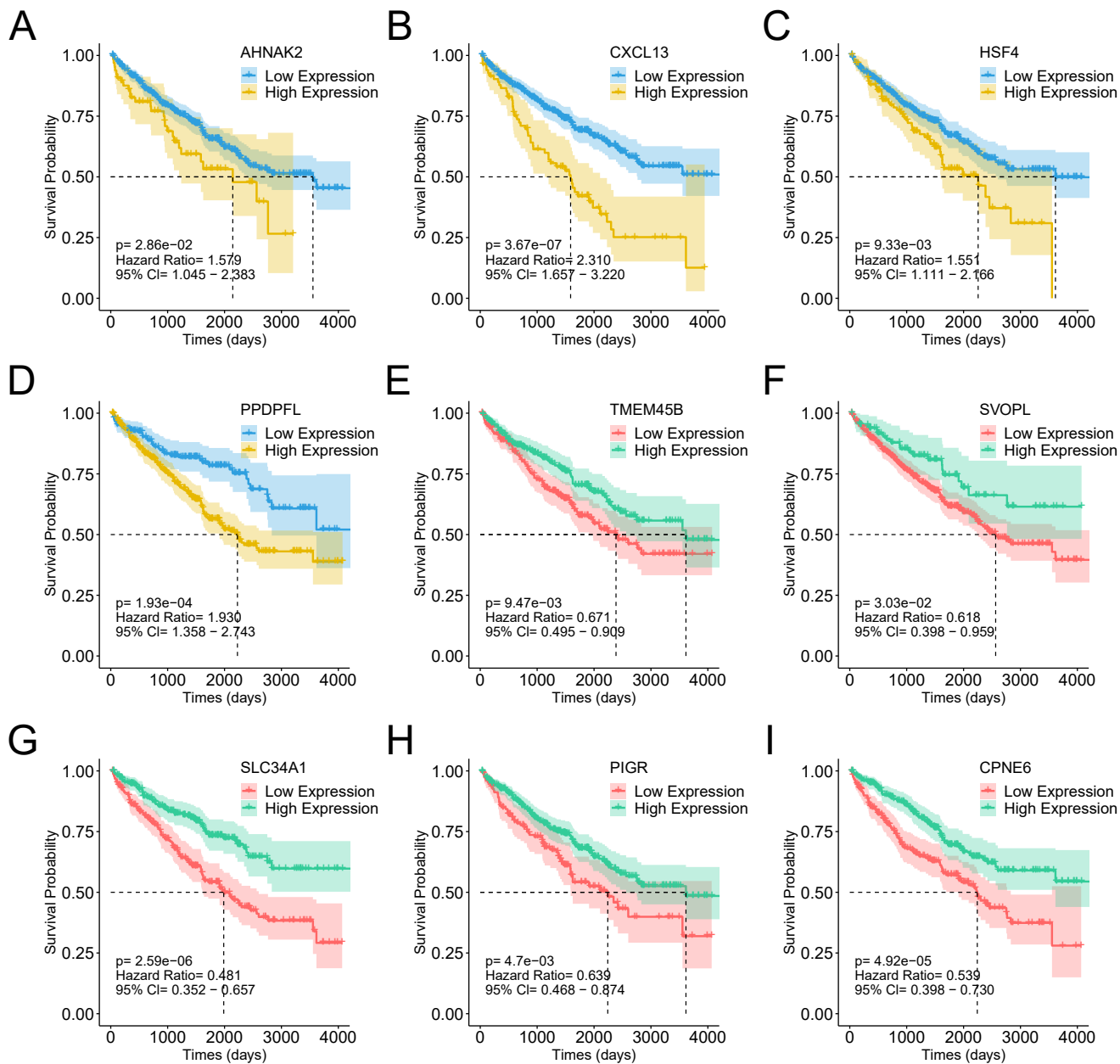

Supplement: Supplementary file 4 — Additional file 4: Figure S1. Kaplan–Meier survival analysis of nine prognosis-related genes used in BSR. BSR, best subset regression. [file 12935_2020_1113_MOESM4_ESM.pdf]

A

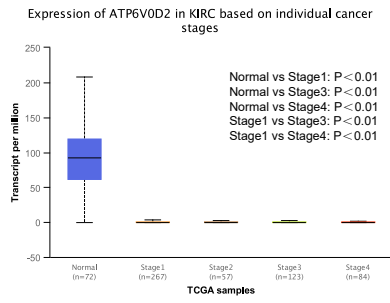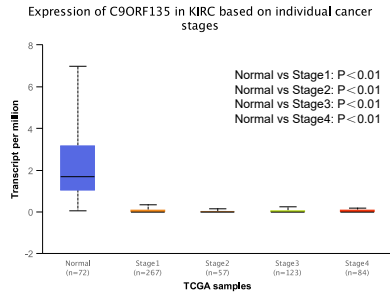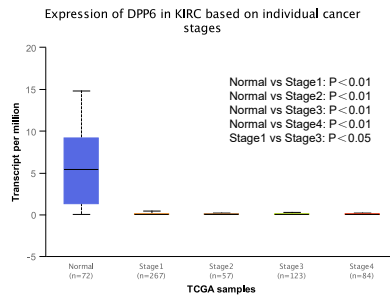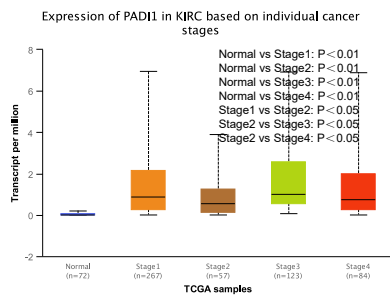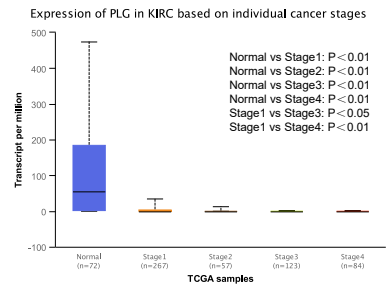

B

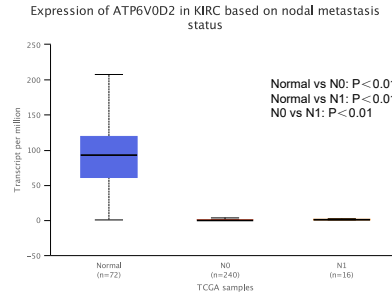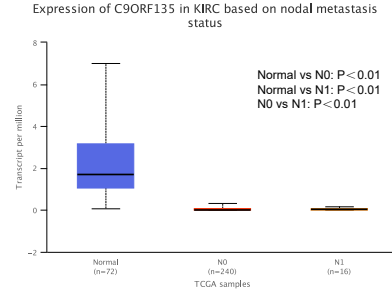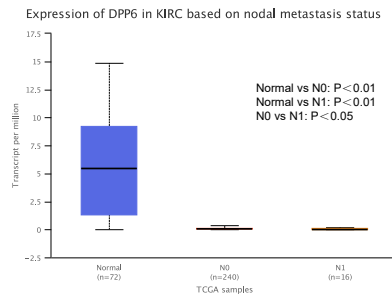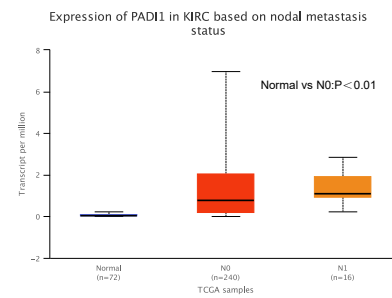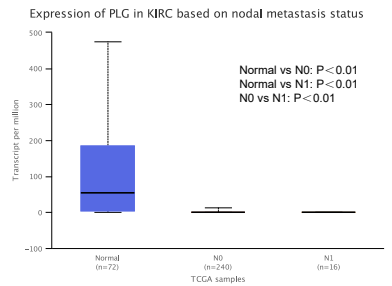

C

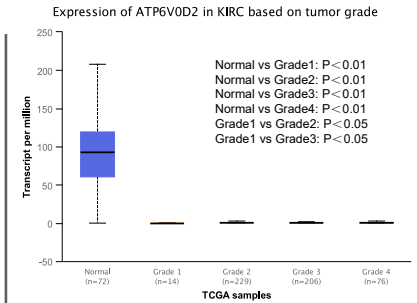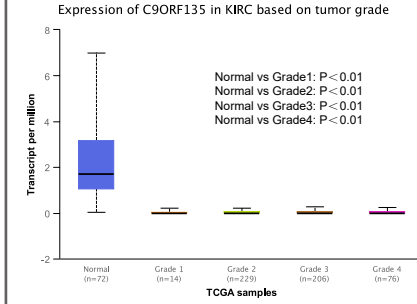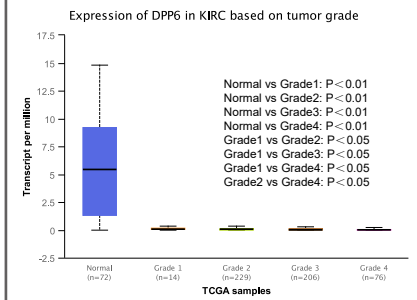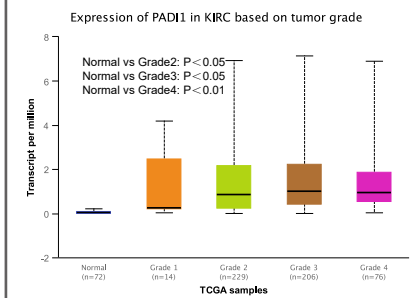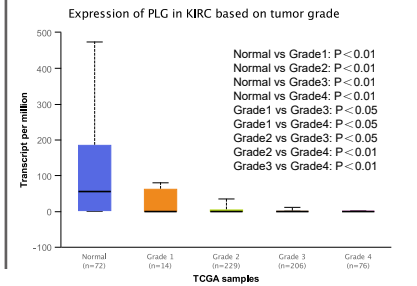

Supplement: Supplementary file 5 — Additional file 5: Figure S2. The expression pattern of the five prognosis-related genes in different AJCC-stage (A), node metastasis state (B) and grade (C) from UALCAN TCGA ccRCC samples. TCGA, The Cancer Genome Atlas. [file 12935_2020_1113_MOESM5_ESM.pdf]

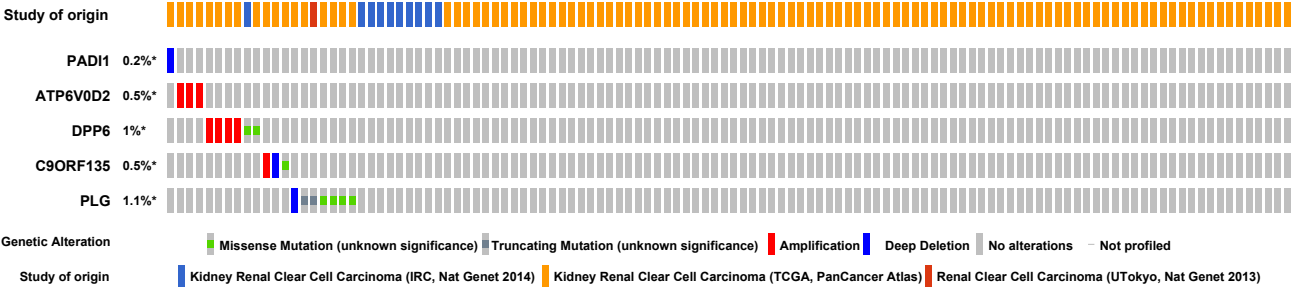

Supplement: Supplementary file 6 — Additional file 6: Figure S3. The type of gene alteration among 628 patietns/696 samples in 3 publicly datasets including TCGA from cBioportal. 23 (3%) patients have gene alternation. [file 12935_2020_1113_MOESM6_ESM.pdf]

**A**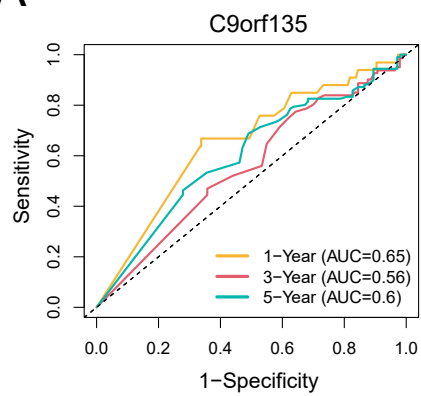**B**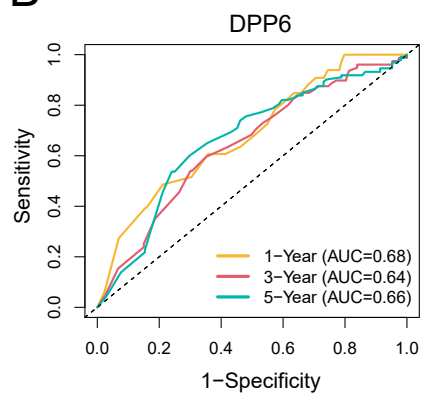**C**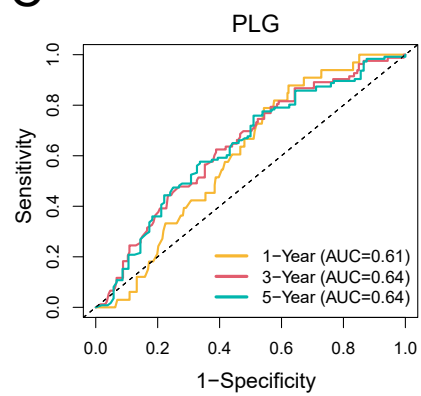**D**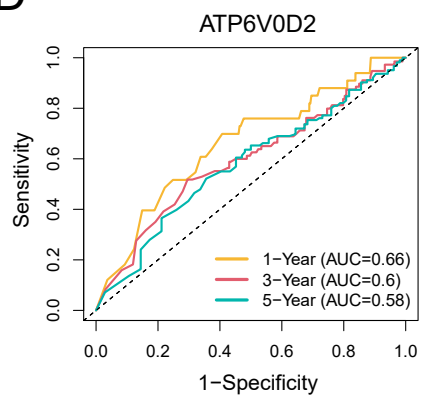**E**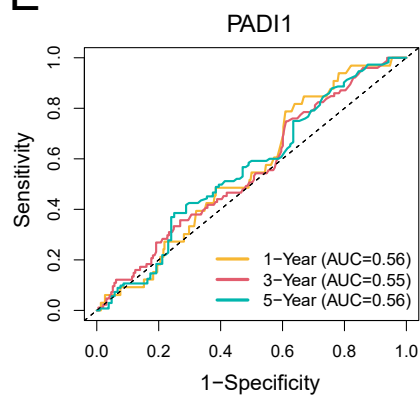

Supplement: Supplementary file 7 — Additional file 7: Figure S4. Time-dependent ROC analysis of the five prognosis-related genes in 1-, 3- and 5-year OS prediction. A C9orf135. B DPP6. C PLG. D ATP6V0D2. E PADI1. ROC, receiver operating characteristic. [file 12935_2020_1113_MOESM7_ESM.pdf]

A

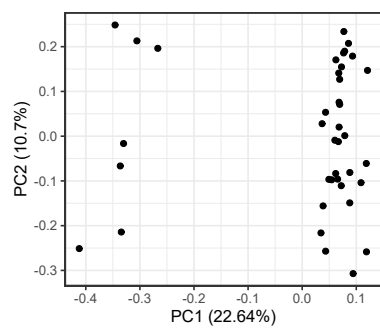

B

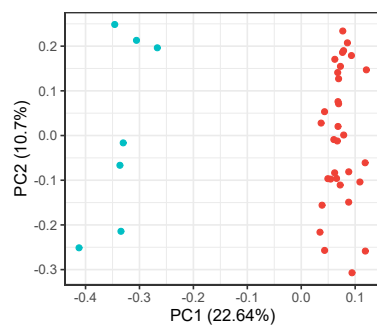

C

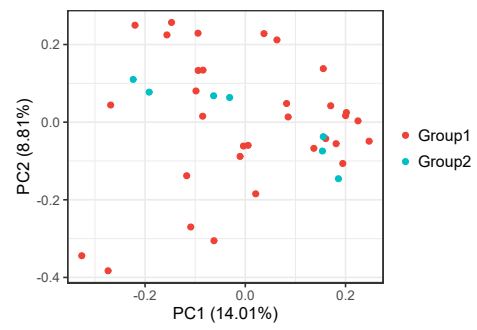

Supplement: Supplementary file 8 — Additional file 8: Figure S5. PCA plot of GSE29609 dataset from GEO. A PCA plot shows that there are two clusters in the dataset, which means the dataset has batch effect. B Colorized the two clusters. C PCA plot of the data after normalization. GEO, Gene Expression Omnibus; PCA, principal component analysis. [file 12935_2020_1113_MOESM8_ESM.pdf]
