# Supplementary material for: Construction of a novel gene-based model for prognosis prediction of clear cell renal cell carcinoma
Source: Cancer Cell Int. 2020 Jan 28;20:27. doi: 10.1186/s12935-020-1113-6 (PMC6986036; doi:10.1186/s12935-020-1113-6)
Supplement: Supplementary file 10 — Additional file 10: Figure S6. Network of GO enriched terms. (A) The color represents the GO terms. (B) The color represents the p-value. The more genes enriched in the terms, the darker the color. Go, Gene ontology. [file 12935_2020_1113_MOESM10_ESM.pdf]

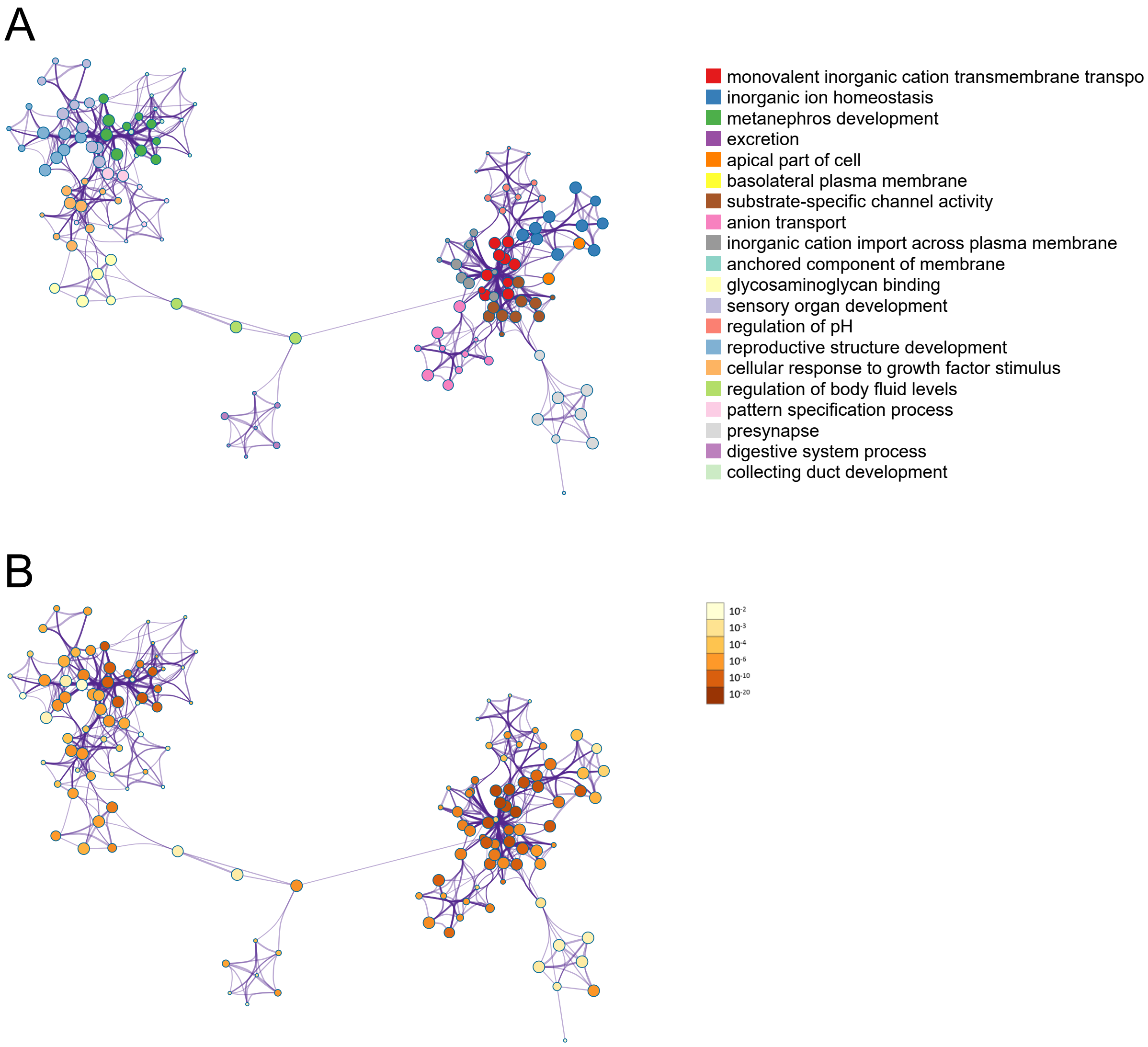
A
monovalent inorganic cation transmembrane transpo
inorganic ion homeostasis
metanephros development
excretion
apical part of cell
basolateral plasma membrane
substrate-specific channel activity
anion transport
inorganic cation import across plasma membrane
anchored component of membrane
glycosaminoglycan binding
sensory organ development
regulation of pH
reproductive structure development
cellular response to growth factor stimulus
regulation of body fluid levels
pattern specification process
presynapse
digestive system process
collecting duct development
B
10^{-2}
10^{-3}
10^{-4}
10^{-6}
10^{-10}
10^{-20}
